# Response and Tolerance of *Macleaya cordata* to Excess Zinc Based on Transcriptome and Proteome Patterns

**DOI:** 10.3390/plants12122275

**Published:** 2023-06-11

**Authors:** Hongxiao Zhang, Linfeng Hu, Xinlong Du, Assar Ali Shah, Baseer Ahmad, Liming Yang, Zhiying Mu

**Affiliations:** 1 College of Agriculture, Henan University of Science and Technology, Luoyang 471000, China; dxl200004@sina.com; 2 College of Biotechnology, Tianjin University of Science and Technology, Tianjin 300457, China; henryhu391@gmail.com; 3 College of Life Sciences, Nanjing Forestry University, Nanjing 210037, China; assaralishah@yahoo.com (A.A.S.); dr.baseerahmadkhan@gmail.com (B.A.); yangliming@njfu.edu.cn (L.Y.); 4 College of Forestry and Biotechnology, Zhejiang Agriculture and Forestry University, Hangzhou 311300, China

**Keywords:** *Macleaya cordata*, transcriptome, proteome, transporter, Zn tolerance, Fe deficiency

## Abstract

*Macleaya cordata* is a dominant plant of mine tailings and a zinc (Zn) accumulator with high Zn tolerance. In this study, *M. cordata* seedlings cultured in Hoagland solution were treated with 200 μmol·L^−1^ of Zn for 1 day or 7 days, and then, their leaves were taken for a comparative analysis of the transcriptomes and proteomes between the leaves of the control and Zn treatments. Differentially expressed genes included those that were iron (Fe)-deficiency-induced, such as vacuolar iron transporter *VIT*, ABC transporter *ABCI17* and ferric reduction oxidase *FRO.* Those genes were significantly upregulated by Zn and could be responsible for Zn transport in the leaves of *M. cordata*. Differentially expressed proteins, such as chlorophyll a/b-binding proteins, ATP-dependent protease, and vacuolar-type ATPase located on the tonoplast, were significantly upregulated by Zn and, thus, could be important in chlorophyll biosynthesis and cytoplasm pH stabilization. Moreover, the changes in Zn accumulation, the production of hydrogen peroxide, and the numbers of mesophyll cells in the leaves of *M. cordata* were consistent with the expression of the genes and proteins. Thus, the proteins involved in the homeostasis of Zn and Fe are hypothesized to be the keys to the tolerance and accumulation of Zn in *M. cordata*. Such mechanisms in *M. cordata* can suggest novel approaches to genetically engineering and biofortifying crops.

## 1. Introduction

Zinc (Zn) is an essential trace element in plants but is also toxic to cells at excessive concentrations. Zn hyperaccumulators are those plants with both a high tolerance for and the ability to accumulate Zn [1]. Therefore, Zn hyperaccumulators have potential use in the phytoremediation of contaminated soils [2]. Furthermore, transgenic approaches can be used to incorporate the traits of hyperaccumulators and biofortify crops [3].

The compartmentation and accumulation of Zn have been investigated in two hyperaccumulators, *Arabidopsis halleri* and *Noccaea caerulescens.* In the hyperaccumulators, most of the Zn was accumulated in epidermal cells and the trichomes of the leaves [4,5]. However, in other studies, Zn primarily accumulated in the vacuoles of mesophyll cells in the leaves of *A. halleri* [6,7]. *Sedum alfredii*, another hyperaccumulator, accumulates 2.9% Zn in the xylem of shoots in a Zn citrate form [6,8]. However, the functions of proteins in plant Zn tolerance and sequestration are unclear.

Most Zn transporters are involved in iron (Fe) transport. For example, in Arabidopsis, iron-regulated transporter 1 and ZIP family transporters are responsible for Fe and Zn uptake [9,10]. Therefore, the cellular utilization of Fe decreases when Zn is in excess, and plants exhibit symptoms of Fe starvation [11]. Excess Zn depressed the activities of Rubisco and PSII in Arabidopsis, which showed symptoms of Fe deficiency [10]. Plants also possess homeostatic mechanisms to compartmentalize heavy metals in different plant tissues to minimize damage [11,12].

*Macleaya cordata* (Willd.) is found in tailing areas, it has a fast growth rate, large biomass, and huge taproots, and has been reported to be a hyperaccumulator for the phytoextraction of uranium- and molybdenum-contaminated soil [13,14]. *M. cordata* also has a good ability to accumulate Zn, mercury, cadmium, lead, and manganese [15,16,17,18,19], indicating that it is a good candidate species for phytoremediation. In addition, we found that *M. cordata* had a very high tolerance to Zn under hydroponic conditions [20], and we also analyzed the oxidative stress response in the roots of *M. cordata* exposed to Zn and Pb [21]. However, little is known about the tolerance and accumulation mechanisms of *M. cordata* to these heavy metals. The objectives of this study were to investigate the mechanisms of response, transport, and tolerance of *M. cordata* to Zn via transcriptome and comparative proteome analyses of the leaves.

## 2. Results

### 2.1. Zn Accumulation in Roots and Shoots of Macleaya cordata

**Figure 1:**
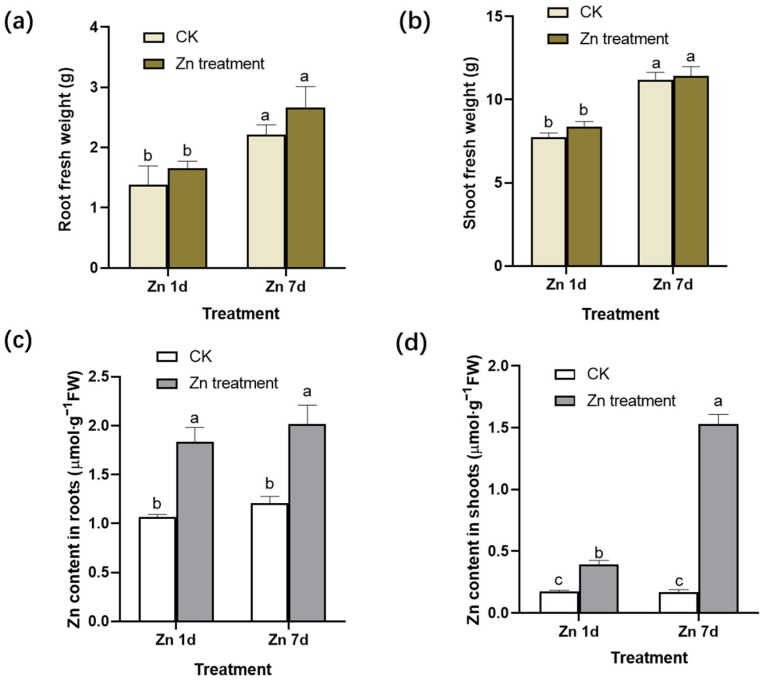
Fresh weight (FW) and zinc (Zn) concentration in (**a**,**c**) the roots and (**b**,**d**) shoots of *Macleaya cordata*. Plants were exposed to control (CK) or 200 μmol·L^−1^ Zn treatment for 1 day (Zn 1d) or 7 days (Zn 7d). Values are the mean ± SE (*n* = 3). Means denoted by different letters are significantly different (*p* < 0.05, Duncan’s test).

After the 200 μmol·L^−1^ Zn treatment for 1 day (Zn 1d) or 7 days (Zn 7d), the fresh weight (FW) in the roots and shoots per plant exhibited no significant change when compared to that of the control (Figure 1a,b). The ratio of the fresh weight to dry weight was 11.2–12.2 of roots or 8.2–9.5 of shoots, and there was no significant difference in the roots or shoots in the present study. However, Zn concentration per gram of fresh weight in the roots and shoots increased significantly (Figure 1c,d). Moreover, with the extension of the treatment time, the increase in Zn concentration in the shoots was more significant than that in the roots, indicating that the leaves of *M. cordata* had a special capacity for Zn accumulation.

### 2.2. H_2_O_2_ Production and Chlorophyll Content in Leaves of Macleaya cordata Exposed to Excess Zn

**Figure 2:**
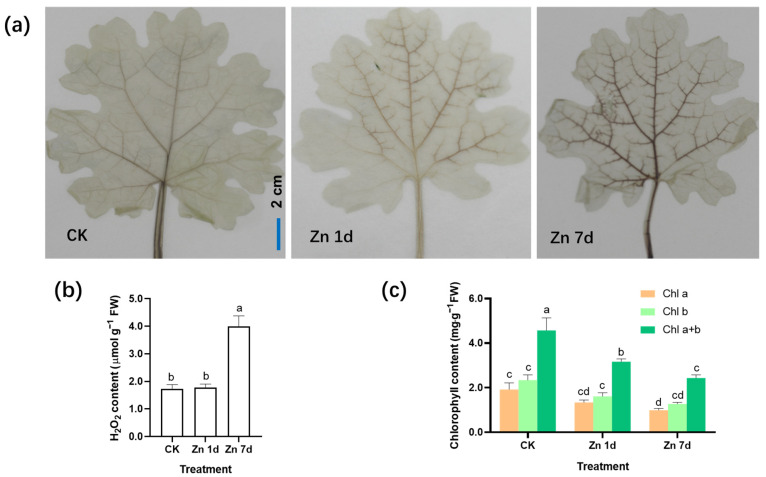
Hydrogen peroxide (H_2_O_2_) production and chlorophyll content in the leaves of *Macleaya cordata* under excess Zn. (**a**) Histochemical location of H_2_O_2_ by 3,3′-diaminobenzidine staining. Bar = 2 cm. (**b**) H_2_O_2_ content and (**c**) chlorophyll content in the leaves of *M. cordata*. Plants were exposed to 200 μmol·L^−1^ Zn for 1 day (Zn 1d) or 7 days (Zn 7d). Values are the mean ± SE (*n* = 3). Means denoted by different letters are significantly different (*p* < 0.05, Duncan’s test). Experiments in (**a**) were repeated at least five times with similar results.

With 3,3′-diaminobenzidine (DAB) staining, reddish brown spots form due to the rapid reaction of H_2_O_2_ with DAB under catalase. The production of H_2_O_2_ was detected by observing the location and intensity of brown spots. The veins of the control plants were lightly stained, whereas the brown color of the leaf veins deepened significantly in the leaves of *M. cordata* under the Zn 7d conditions (Figure 2a). The concentrations of H_2_O_2_ in the leaves assayed using spectrophotometry were consistent with those of histochemical detection via DAB staining (Figure 2b). Therefore, oxidative stress in mesophyll cells increased after Zn treatment for 7 d. In addition, the contents of chlorophyll a and b in the leaves of *M. cordata* decreased significantly under the Zn treatment (Figure 2c).

### 2.3. Transcriptomic and Proteomic Analysis Overview

**Figure 3:**
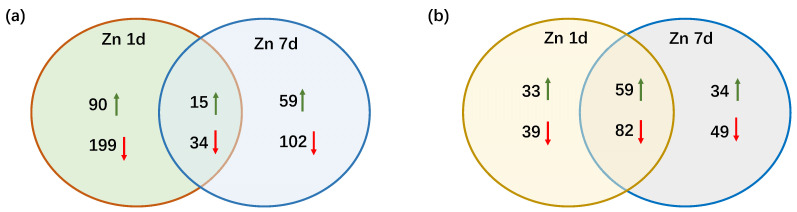
Venn diagrams showing the numbers of (**a**) differentially expressed genes and (**b**) differentially expressed proteins in the leaves of *Macleaya cordata*. Upward green arrow shows an increase and the downward red arrow shows a decrease in the expression of genes or proteins in the leaves of *M. cordata* exposed to 200 μmol·L^−1^ Zn for 1 day (Zn 1d) or 7 days (Zn 7d).

In the transcriptome of *M. cordata* leaves, 32,485 non-redundant transcripts were annotated, and a total of 499 differentially expressed genes (DEGs) were screened for significant differential expression with |log_2_(fold change)| > 1 between the two sample sets (Zn 1d vs. CK and Zn 7d vs. CK). An overview of the numbers of DEGs in Zn 1d and Zn 7d is shown in Figure 3a. Ninety DEGs were upregulated only in Zn 1d, and 59 DEGs were upregulated only in Zn 7d. However, there were almost twice as many downregulated DEGs as upregulated DEGs in either Zn 1d or Zn 7d. Moreover, fifteen DEGs were upregulated and thirty-four DEGs were downregulated in both Zn 1d and Zn 7d, respectively.

After the MS/MS raw data of the proteome were searched against the *M. cordata* transcriptome, a total of 296 differentially expressed proteins (DEPs) were screened, and significant differential expressions between the Zn and control treatments of 1.5-fold (up) or 0.67-fold (down) change were found. Venn diagrams of the protein expression in response to the Zn 1d and Zn 7d treatments are shown in Figure 3b. There were 92 DEPs that were upregulated in Zn 1d and 93 DEPs that were upregulated in Zn 7d. The number of downregulated DEPs was slightly greater than that of upregulated DEPs in either the Zn 1d or Zn 7d treatments, and there were 59 upregulated and 82 downregulated DEPs in both the Zn 1d and Zn 7d treatments.

### 2.4. Characteristic of Transporter Genes in Leaves of Macleaya cordata

**Figure 4:**
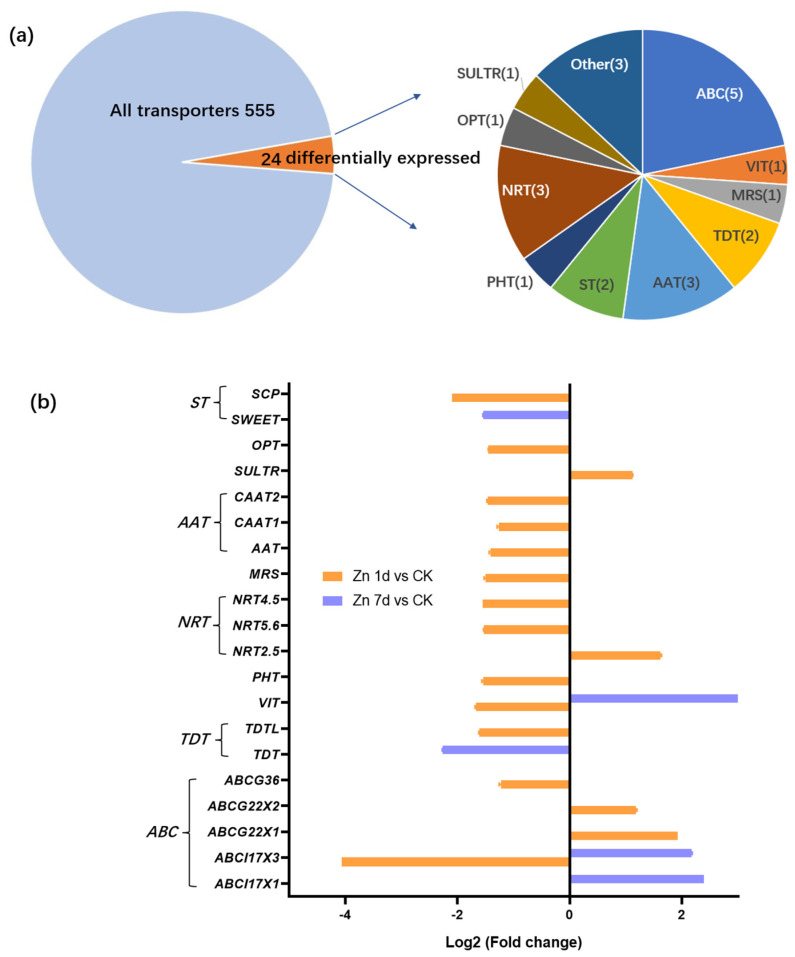
Identification and expression levels of putative Zn transporters in the leaves of *Macleaya cordata*. (**a**) Proportions of the identified transporters. (**b**) Expression levels of the identified transporters. Plants were exposed to 200 μmol·L^−1^ Zn for 1 day (Zn 1d) or 7 days (Zn 7d). Expression levels of transporters were based on Log2 (Fold change) (*n* = 3, padj < 0.05). *ABCI17X1* and *ABCI17X3*: ATP-biding cassette (ABC) transporter family I members; *ABCG22X1*, *ABCG22X2*, *ABCG36*: ABC transporter family G members; *TDT(L)*: tonoplast dicarboxylate transporter (like); *VIT*: vacuolar iron transporter; *PHT*: phosphate transporter; *NRT2.5, 5.6, 4.5*: nitrate transporter family members; *MRS*: magnesium transporter; *AAT*: amino acid transporter; *CAAT*: cationic amino acid transporter; *SULTR*: sulfate transporter; *OPT*: oligopeptide transporter; *ST*: sugar transporter; *SWEET*: bidirectional sugar transporter; *SCP*: sugar carrier protein.

A total of 555 non-redundant transporters were identified from the transcriptome of *M. cordata* leaves. Among the transporters, 24 transporter genes were identified as DEGs in the leaves of *M. cordata* under the Zn treatment (Figure 4a). The genes were mainly categorized into ATP-binding cassette transporters (ABC, 5 of 24), amino acid transporters (AAT, 3 of 24), nitrate transporters (NRT, 3 of 24), two tonoplast dicarboxylate transporters (TDT), two sugar transporters (ST), a phosphate transporter (PHT), a vacuolar iron transporter (VIT), a sulfate transporter (SULTR), an oligopeptide transporter (OPT) and three other transporters (Figure 4a). The expression change in the transporters under the Zn treatment was analyzed (Figure 4b). The Zn treatment downregulated 15 of the 20 transporters; however, four ABC transporter genes were upregulated in Zn 1d or 7d. Five transporter genes, including VIT, ABCI17X1, and ABXI17X3, were upregulated by the Zn 7d treatment. Significantly, both VIT and ABCI17X3 genes were downregulated in the Zn 1d treatment and were upregulated in the Zn 7 d treatment.

### 2.5. Differentially Expressed Genes Involved in Response and Tolerance of Macleaya cordata to Zn

**Figure 5:**
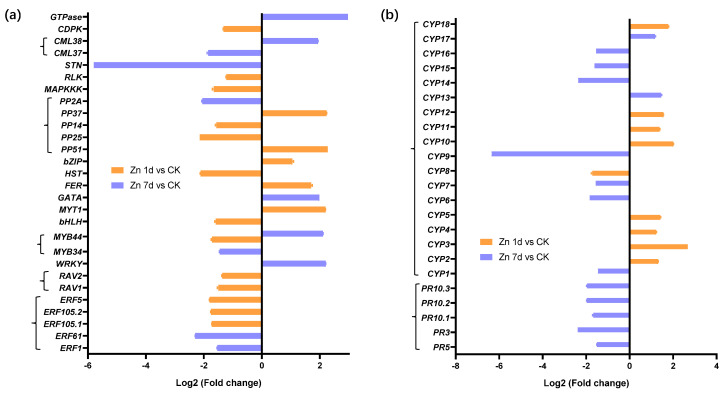
The expression levels of genes involved in (**a**) signal transduction and (**b**) stress response in the leaves of *Macleaya cordata*. Plants were exposed to 200 μmol·L^−1^ Zn for 1 day (Zn 1d) or 7 days (Zn 7d). The expression levels of genes were based on Log2 (Fold change) (*n* = 3, padj < 0.05). *ERF*: ethylene-responsive transcription factor; *RAV*, *WRKY*, *MYB*, *bHLH*, *GATA*, *bZIP*: transcription factors; *MYT*: myelin transcription factor; *FER*: iron-deficiency-induced transcription factor; *HST*: heat stress transcription factor; *PP*: phosphatase; *MAPKKK*: mitogen-activated protein kinase kinase kinase; *RLK*: receptor-like protein kinase; *STN*: serine/threonine protein kinase; *CML*: calcium-binding protein; *CDPK*: calcium-dependent protein kinase; *CYP*: cytochrome P450; *PR*: pathogenesis-related protein.

Twenty-eight DEGs were involved in signal transduction (Figure 5a), and eighteen appeared in the Zn 1d treatment. With the exception of the transcription factor of the MYB44 gene, which appeared in both Zn 1d and Zn 7d, 27 DEGs were upregulated in either the Zn 1d or Zn 7d conditions, and 19 of the 28 DEGs were downregulated by Zn. Among those DEGs, the expression of the serine/threonine protein kinase (STN) gene decreased the most, whereas GTPase increased the most, and both appeared in the Zn 7d treatment. Five genes, including GTPase, calcium-binding protein (CML), WRKY, MYB44, and GATA transcription factors, were upregulated in the Zn 7d treatment, and five other genes, including those for myelin (MYT1), bZIP, and iron deficiency response (FER) transcription factors and two phosphatases (PP37 and PP51), were upregulated in the Zn 1d treatment. Other DEGs involved in signal transduction were downregulated by the Zn treatment. Furthermore, 18 DEGs had at least an annotation for cytochrome P450 (CYP) from NCBI (nr), Swiss-port, GO, COG, KOG, or KEGG, and five pathogenesis-related protein (PRs) were downregulated in the Zn 7d treatment (Figure 5b).

**Figure 6:**
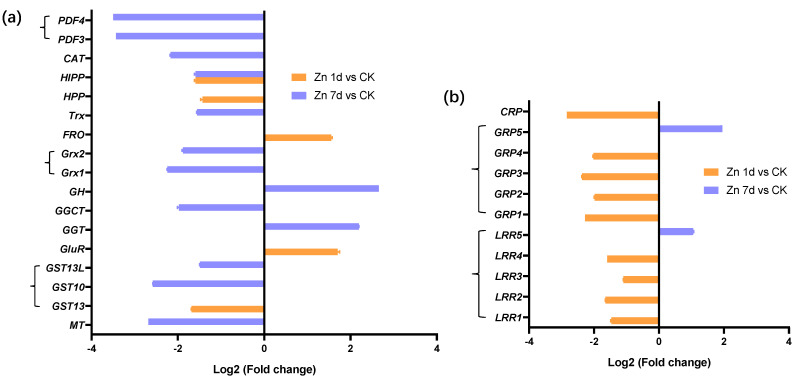
Expression levels of genes involved in (**a**) cysteine metabolism and associated with (**b**) cell wall structural proteins in the leaves of *Macleaya cordata*. Plants were exposed to 200 μmol·L^−1^ Zn for 1 day (Zn 1d) or 7 days (Zn 7d). The expression levels of the genes were based on Log2 (Fold change) (*n* = 3, padj < 0.05). *MT*: metallothionein; *GST*: glutathione S-transferase; *GluR*: glutamate receptor; *GGT*: gamma-glutamyltranspeptidase; *GGCT*: gamma-glutamylcyclotransferase; *GH*: glutathione hydrolase; *Grx*: glutaredoxin; *FRO*: ferric reduction oxidase; *Trx*: thioredoxin; *HPP*: metal-ion-binding protein; *HIPP*: heavy-metal-associated isoprenylated plant protein; *CAT*: catalase; *PDF*: defensin-like protein; *LRR*: leucine-rich repeat receptor protein kinase; *GRP*: glycine-rich cell wall structural protein; *CRP*: chitin recognition protein.

Seventeen DEGs were involved in cysteine metabolism (Figure 6a), but only five DEGs appeared in the Zn 1d treatment. Among them, four DEGs were upregulated by Zn, and glutathione hydrolase (GH) was the most upregulated in the Zn 7d treatment. Two DEGs of defensin-like proteins (PDF3 and PDF4) were the most downregulated in the Zn 7d treatment. Metallothionein (MT) and glutathione S-transferase (GST10) were also most downregulated in the Zn 7d treatment. A heavy-metal-associated isoprenylated plant protein (HIPP) gene was downregulated in both the Zn 1d and 7d treatments. Eleven DEGs involved in cell wall structural proteins were downregulated in the Zn 1d treatment, and only leucine-rich repeat receptor protein kinase (LRR5) and a glycine-rich cell wall structural protein (GRP5) were upregulated in the Zn 7d treatment (Figure 6b).

### 2.6. Validation of DEGs by Quantitative Real-Time PCR (qRT-PCR)

**Figure 7:**
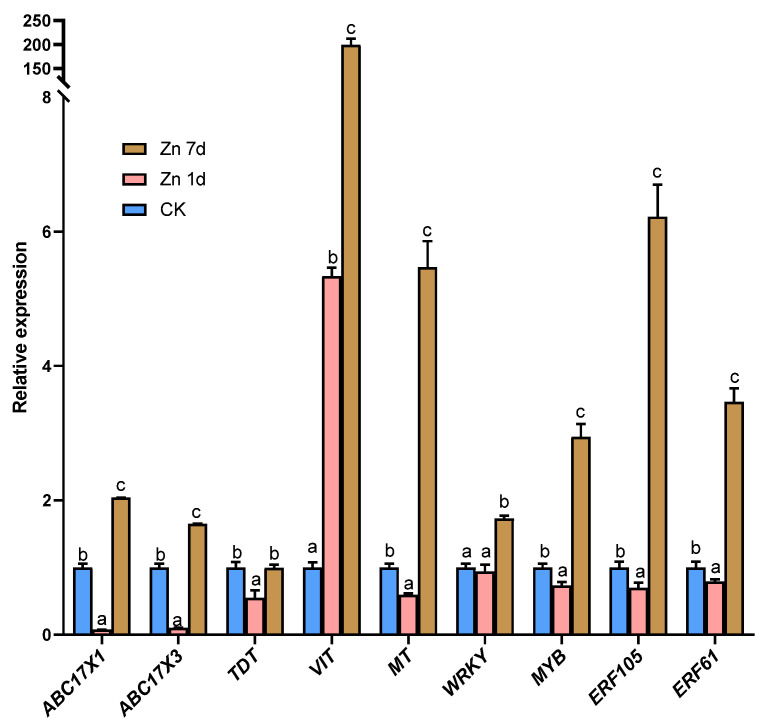
The relative expression level of genes in the leaves of *Macleaya cordata* via quantitative real-time PCR. Plants were exposed to 200 μmol·L^−1^ Zn for 1 day (Zn 1d) or 7 days (Zn 7d). The relative expression levels of genes denoted using different letters indicate significant differences (*p* < 0.05, Duncan’s test).

The expression of nine DEGs from the transcriptomic analysis was verified through the expression levels according to qRT-PCR. The relative expressions of ABC17X1, ABC17X3, TDT, WRKY, and MYB genes were consistent with the results of the transcriptome; however, VIT gene expression increased in the Zn 1d treatment according to qRT-PCR, in contrast to the results in the transcriptome (Figure 7). In addition, the relative gene expression of MT, ERF105, and ERF61 increased significantly in the Zn 7d treatment, which is in contrast to the results of the transcriptome.

### 2.7. Proteomic Profiling of Leaves in Macleaya cordata under Zn Treatment

**Figure 8:**
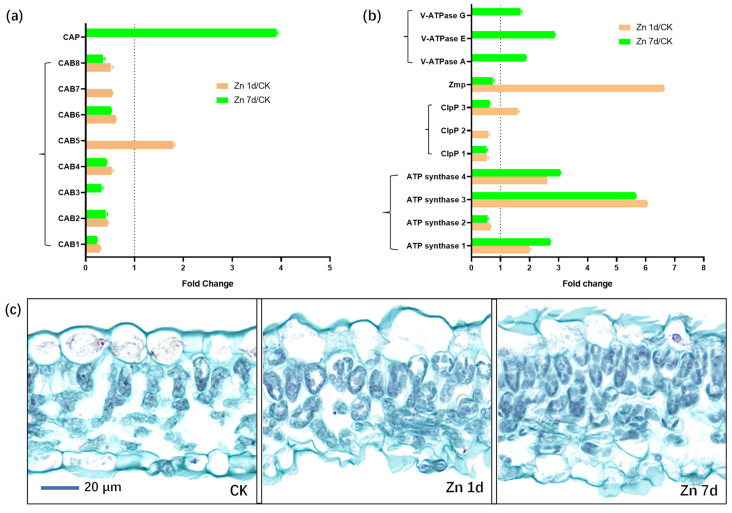
The expression levels of the proteins involved in (**a**) chlorophyll and (**b**) ATP metabolism, and (**c**) cytochemical characteristics of mesophyll cells in leaves of *Macleaya cordata*. Plants were exposed to 200 μmol·L^−1^ Zn for 1 day (Zn 1d) or 7 days (Zn 7d). Expression levels of proteins were based on fold change (*p* < 0.05, Student’s *t*-test). Paraffin section experiments were repeated at least three times with similar results. CAB: chlorophyll a/b-binding protein; CAP: chlorophyll apoprotein; ClpP: ATP-dependent protease; ZMP: ATP-dependent zinc metalloprotease; V-ATPase: vacuolar-type ATPase.

Eight DEPs were identified as chlorophyll a-b binding proteins (CABs), but only CAB5 was upregulated in the Zn 1d treatment (Figure 8a). Moreover, the chlorophyll apoprotein (CAP) gene was also upregulated in the Zn 7d treatment. Eleven DEPs were involved in ATP metabolism (Figure 8b), and three of the four ATP synthase genes were upregulated in both the Zn 1d and 7d treatments. However, three vacuolar-type ATPase (V-ATPase) genes were upregulated in the Zn 7d treatment, and the genes of an ATP-dependent Clp protease (ClpP3) and zinc metalloprotease (ZMP) were also upregulated in the Zn 1d treatment. The morphology of mesophyll cells was determined via paraffin sectioning with safranin and fast green staining (Figure 8c). Compared with the control, the number of mesophyll cells increased significantly under the Zn treatments, and the degree of increase was most pronounced in the Zn 7d treatment.

**Figure 9:**
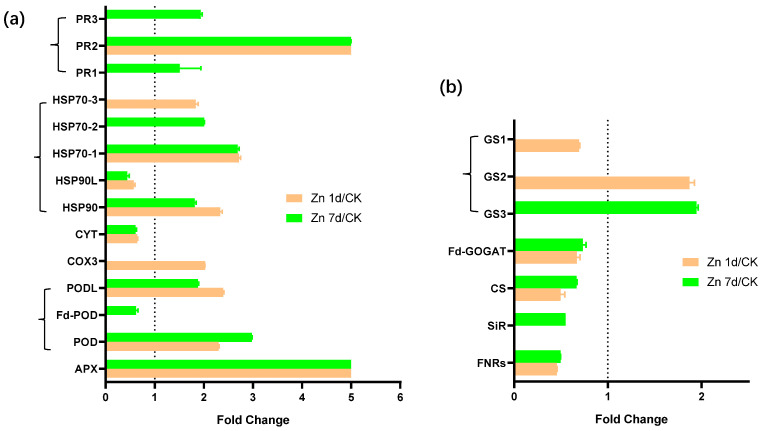
The expression levels of the proteins involved in (**a**) stress response and (**b**) sulfur metabolism in the leaves of *Macleaya cordata*. Plants were exposed to 200 μmol·L^−1^ Zn for 1 day (Zn 1d) or 7 days (Zn 7d). Expression levels of proteins were based on fold change (*p* < 0.05, Student’s *t*-test). APX: ascorbate peroxidase; POD: peroxidase; COX: cytochrome c oxidase; CYT: cytochrome complex; HSP: heat shock protein; FNR: ferredoxin–NADP reductase; SiR: sulfite reductase; CS: cysteine synthase; Fd-GOGAT: ferredoxin-dependent glutamate synthase; GS: glutamine synthetase.

Fourteen DEPs were stress-response-related proteins, and most, including one APX, two of three PODs, three of the four heat shock proteins (HSPs), and three PRs, were upregulated by Zn (Figure 9a). Seven DEPs were involved in sulfur metabolism (Figure 9b), but only two glutamine synthetase genes (GS2 and GS3) were upregulated by Zn. However, ferredoxin–NADP reductase (FNR), sulfite reductase (SiR), cysteine synthase (CS), ferredoxin-dependent glutamate synthase (Fd-GOGAT), and GS1 were downregulated by Zn.

## 3. Discussion

### 3.1. Increase in Zn Concentration and H_2_O_2_ Production in Leaves of Macleaya cordata Exposed to Zn

In a previous study, compared with the control, 200 μmol·L^−1^ Zn for 7 days significantly inhibited the root length and shoot height of *M. cordata* [21], and the same growth inhibition also appeared in the present study. Compared to the control, there was no significant difference in the fresh weight per plant under the Zn 1d and Zn 7d conditions (Figure 1a,b). Moreover, the Zn concentration in the roots and shoots of *M. cordata* clearly increased under excess Zn (Figure 1c,d). With extended treatment time, the Zn concentration in the roots and shoots of *M. cordata* increased further, but the proportion of Zn increase in shoots was much greater than that in the roots in the Zn 7d treatment. This result was consistent with our previous studies [20,21]. When comparing Zn concentrations in solutions and in plants, we found that only a small amount of Zn in the solutions was transported to the plants, and most of them were transported and accumulated in the shoots of *M. cordata* during the Zn treatments (Figure 1c,d). Thus, the tolerance to Zn in *M. cordata* is higher in the leaves than in the roots.

In plants exposed to excess Zn, H_2_O_2_ acts as both a signal molecule and an indicator of oxidative stress [22,23]. The production of H_2_O_2_ in the leaves of *M. cordata* increased significantly with the extension of Zn treatment tie (Figure 2a,b). However, the content of chlorophyll a and b decreased significantly with the Zn treatment (Figure 2c). The results indicated that oxidative stress occurred in mesophyll cells exposed to excess Zn.

### 3.2. An Fe Deficiency Signal Regulates Expression of Transporter Genes in Macleaya cordata

Vacuolar sequestration is a strategy that safely stores excess metal ions to reduce toxicity to the cells [24]. Vacuolar iron transporter (VIT) is a transporter that transports cytoplasmic Fe ions as well as Zn along with Fe into vacuoles [25]. However, when there is excess Zn in plants, cellular Fe utilization decreases, and plants exhibit Fe deficiency signals [23]. Shinozaki and Yoshimoto [11] found that VITs can help maintain Fe and Zn homeostasis in cells rather than allocating Fe to vacuoles. The knockout of *OsVIT2* causes an increase in Fe accumulation in rice grains [26]. In this study, according to both the transcriptome and qRT-PCR results, the VIT gene in the leaves of *M. cordata* was significantly upregulated in the Zn 7d treatment (Figure 4b and Figure 7). It was hypothesized that VIT transported both Zn and Fe to vacuoles and, as a result, Fe deficiency developed in the leaves of *M. cordata* under excess Zn.

ABC transporters have crucial roles in the pathways of plant secondary metabolites and responses to environmental stress. In this study, among ABC transporters, two I family numbers, *ABCI17X1* and *ABCI17X3*, were upregulated in the Zn 7d treatment (Figure 4b and Figure 7), and two G family members, *ABCG22X1* and *ABCG22X2*, were upregulated in the Zn 1d treatment (Figure 4b). Two Arabidopsis ABC transporters, *AtABCI10* and *AtABCI11*, are significantly induced by Fe deficiency and regulate chloroplast biogenesis and metal homeostasis [27]. The transporter OsABCI7, located on the thylakoid membrane of rice, can regulate intracellular reactive oxygen species (ROS) homeostasis and maintain the stability of the thylakoid membrane [28]. According to Kuromori et al. [29], Arabidopsis AtABCG25 is a plasma membrane exporter of ABA in the ABA signaling pathway. In addition, AtABCG40, located on the plasma membrane, is a pump that excludes lead as well as other toxic compounds from the cytoplasm [30,31]. Therefore, it was hypothesized that (1) the increase in the expression of ABCI17 located on the thylakoid membrane transported Fe to chloroplasts in order to synthesize chlorophyll, and (2) the increase in the expression of ABCG22 located on the plasma membrane was important in transporting Zn and other compounds to apoplasts in the leaves of *M. cordata* exposed to excess Zn.

Carboxylic acids, such as malate, citrate, and fumarate, can chelate metallic nutrients and toxic heavy metals [32]. A high concentration of citrate has been detected in the xylem of the hyperaccumulator *S. alfredii*, and the amount of citrate increased significantly with an increase in Zn concentration [6,33]. In addition, in mutants of tonoplast dicarboxylate transporters (TDTs), leaf citrate and malate levels decreased in Arabidopsis [34,35]. In this study, two TDT transporter genes, *TDT* and *TDTL*, were downregulated (Figure 4), which was attributed to reductions in carboxylic acids. Peptide transporter AtOPT3 loads Fe into the phloem for Fe redistribution from mature to developing tissues in Arabidopsis [36]. Moreover, *OPT3* is highly induced by Fe deficiency in the vascular systems of *N. caerulescens* [37]. In this study, *OPT3* was downregulated in the Zn 1d treatment (Figure 4b), thereby reducing long-distance Fe transport in *M. cordata*.

Concerning nitrate transporter family members, *NRT2.5* was upregulated, but *NRT4.5* and *NRT5.6* were downregulated in the Zn 1d treatment (Figure 4b). Excess Zn increases the expression of Arabidopsis *AtNRT1.1* to promote nitrate absorption and Zn transport [38]. However, in mutants of *AtNRT1.1*, Zn accumulation in Arabidopsis decreases, as does the inhibition of photosynthesis caused by Zn stress [38]. In addition, a magnesium transporter, a phosphate transporter, two sugar transporters, and three amino acid transporters were downregulated in Zn 1d in the present study (Figure 4b), although the roles in Zn transport in *M. cordata* were unclear.

### 3.3. Macleaya cordata Regulate Zn Tolerance by Multiple Signal Pathways

*Macleaya cordata* has a high tolerance to Zn [19,20,21]. In this study, ten of twenty-four genes associated with signal transduction were upregulated in the leaves by Zn. The DEGs of MYT, FER, bZIP, and two PPs were upregulated in the Zn 1d treatment, whereas the DEGs of WRKY, MYB44, GATA, CML, and GTPase were upregulated in the Zn 7d treatment (Figure 5a). In addition, 19 genes involved in signal pathways were downregulated in the Zn 1d or Zn 7d treatments. The WRKY transcription factors have a novel Zn-chelating DNA-binding domain, and several WRKYs are upregulated in response to the H_2_O_2_ treatment in *A. thaliana* [39]. In Pepper, WRKYs are also upregulated by Cd and H_2_O_2_ stress [40], and the overexpression of *ThWRKY* improves the Cd tolerance of *Saccharomyces cerevisiae* [40,41]. In this study, *STN* was downregulated almost six times in the Zn 7d treatment (Figure 5a). In rice, the loss-of-function of *OsSTN8* suppresses photosystem II phosphorylation [42]. Therefore, there were multiple signal pathways that regulated transport or antioxidant protection during the Zn treatment.

Cell wall structural proteins, including LRR, GRP, and CRP, function during signal transduction in higher plants [43,44,45,46], and in particular, LRRs conduct cell wall signals to regulate plant growth and stress tolerance [44]. In the present study, most cell wall structural protein genes were downregulated in the Zn 1d treatment; only *LRR5* and *GRP5* were upregulated in the Zn 7d treatment (Figure 6b).

The FRO genes encode ferric chelate reductase, which is responsible for the reduction of Fe, and it is mainly expressed in the cytoplasm to transport Fe to chloroplasts in leaves [47,48]. The overexpression of *AtFRO2* increases Arabidopsis tolerance to low iron [47]. The FRO gene was upregulated in the Zn 1d treatment (Figure 6a) and thus could regulate the balance between excess Zn and Fe deficiency. PDFs are cysteine-rich peptides that have a range of biological functions, including defending against heavy metal stress [49]. The overexpression of *AtPDF1.1* increases the sequestration of Fe in Arabidopsis leaves and consequently activates an iron-deficiency-mediated response via the ethylene signal pathway [49]. The Grxs and Trxs are small oxidoreductases that have roles in the biogenesis of iron–sulfur clusters. In Arabidopsis, the expression of *Grxs* and *Trxs* is downregulated via the hydrolysis of GSH [50,51]. Two PDFs, two *Grxs* and one *Trx* were downregulated in the Zn 7 d treatment (Figure 6a), which might induce Fe deficiency and GSH degradation in the leaf cells of *M. cordata* exposed to excess Zn. Ferredoxins (Fds), which are iron–sulfur proteins, have crucial roles in photosynthetic electron transport and are especially important for energy conservation [52]. In this study, the decrease in the Fd-containing proteins Fd-GOGAT and FNR could be caused by Fe deficiency (Figure 9b).

When plants respond to environmental stress, GSH is the most abundant nonprotein thiol [32]. Sulfur and GSH metabolism are important in plant tolerance to heavy metals [53,54,55]. In this study, most DEGs involved in cysteine and GSH metabolism were downregulated by Zn. The exceptions were *FRO* and *GluR*, which were upregulated in the Zn 1d treatment, and GH and GGT were upregulated in the Zn 7d treatment (Figure 6a). The GGTs and GHs are enzymes that hydrolyze GSH, which releases glutamates and cysteines to some acceptors [56]. Wound-induced electrical signals, cytoplasmic Ca^2+^ concentration, and glutamate can induce *GluR* expression in Arabidopsis [57,58]. In the leaves of *M. cordata* under the Zn treatment, the upregulated DEGs of *GluR*, *GH*, and *GGT* and the downregulated DEGs of *Grx* and *Trx* indicated that the hydrolysis of GSH increased to provide additional cysteines and glutamates (Figure 6a). The increase in cysteines could be used to synthesize other metal-binding proteins, such as MTs (Figure 4b and Figure 7). The HPPs and HIPPs are a group of metallochaperones, which play important roles in metal homeostasis [59]. In this study, *HPP* and *HIPP*, which were downregulated by the Zn treatment, could be consumed to maintain Zn-Fe homeostasis (Figure 6). The enzyme GS catalyzes the conversion of glutamate into glutamine, and it is also a key enzyme involved in nitrogen assimilation during the development of wheat [60]. In this study, the increase in DEPs of GS2 and GS3 (Figure 9b) further confirmed the degradation of GSH in the leaves of *M. cordata* under Zn treatment.

Excess Zn induced H_2_O_2_ production and antioxidant defense in the leaves of *M. cordata* (Figure 2a,b). The DEPs involved in antioxidant defense, including two PODs and one APX, were significantly upregulated by Zn (Figure 9a). Those proteins likely had active roles in removing H_2_O_2_ in the leaves of *M. cordata* exposed to excess Zn (Figure 2b). Moreover, four HSPs and three PRs were upregulated in the Zn 1d or 7d treatments. An increase in PRs and HSPs was also observed in Cu-treated [54,61] or H_2_O_2_-treated rice [55].

### 3.4. Macleaya cordata Regulate Zn and Fe Homeostasis by Chlorophyll and ATP Metabolism

Chloroplasts are the major sink in terms of Fe in leaves [62,63]. Fe is also an essential cofactor in chlorophyll biosynthesis enzymes, and low Fe leads to a decrease in chlorophyll synthesis [11]. In *M. cordata*, most CABs (except CAB5) were downregulated by Zn, and a CAP was significantly upregulated in the Zn 7d treatment (Figure 8a). A decrease in the content of chlorophylls was consistent with CAB expression in the leaves of *M. cordata* under Zn treatments (Figure 2c). Chlorophyll is bound to different chlorophyll-binding proteins, which then become the core complexes of the two photosystems [64]. Chlorophyll content in the leaves affects the stabilization and expression of CAPs [65]. The expression of CAB and CAP influences chlorophyll biosynthesis in *Camellia sinensis* [66] and Arabidopsis [64].

Chlorophyll absorbs light energy via photosystems and ultimately provides energy for plant growth and other biological processes, including ion transport and antioxidant protection. In this study, DEPs involved in ATP metabolism, including three ATP synthases and three V-ATPases, were upregulated by Zn (Figure 8b). The V-ATPases have vital roles in intracellular acidic compartments and can biosynthesize ATP in yeast vacuoles [67]. In addition, Zn transport in yeast vacuolar membranes requires V-ATPase [68]. The ATP-dependent ClpP and Zmp are the major proteases in chloroplast protein homeostasis [69]. The protease ClpP is involved in Fe homeostasis in Arabidopsis leaves, and the loss of *Clp* results in decreases in FROs in chloroplasts [70]. On the other hand, the degradation products of chloroplasts by proteases are beneficial for synthesizing new mesophyll cells, which can ultimately regulate the numbers of mesophyll cells and Fe homeostasis in chloroplasts. In addition, Zmp has critical roles in the biogenesis of thylakoid membranes [69]. In this study, ClpP 3 and Zmp were upregulated in the Zn 7d treatment (Figure 8b), which could explain the increase in the number of chloroplasts and mesophyll cells in the leaves of *M. cordata* (Figure 8c).

## 4. Materials and Methods

### 4.1. Plant Material and Hydroponic Culture

Seeds of *M. cordata* were collected from the tailings of Huaguoshan Town, Luoyang City, China (Lat. 39°19′ N, Long. 111°53′ E). The seeds were germinated in vermiculite, and then eight seedlings were cultured with a 2.5 L plastic vessel containing Hoagland nutrient solution (1 mM KH_2_PO_4_, 1 mM KNO_3_, 1 mM Ca(NO_3_)_2_, 1 mM MgSO_4_, 20 μM Fe-EDTA, 46 μM H_3_BO_3_, 9 μM MnCl_2_, 0.76 μM ZnSO_4_, 0.32 μM CuSO_4_, and 0.11 μM H_2_MoO_4_) under controlled conditions (14 h day length with photosynthetically active radiation of 400 μmol m^−2^ s^−1^ and 25/20 °C day/night temperatures). The solution pH was adjusted to 5.3, with the renewal of the nutrient solution every 2 days. Uniform 20-day-old seedlings with four leaves were treated with 200 μmol·L^−1^ Zn for 1 day (Zn 1d) or 7 days (Zn 7d). Zn was applied as ZnSO_4_·7H_2_O. The control plants were cultivated in a complete Hoagland solution, with a minimum of 0.76 μmol L^−1^ Zn (CK). After Zn exposure for 1 d or 7 d, the shoots and roots of *M. cordata* were, respectively, collected for the determination of Zn content, and the second youngest leaves were separated for the detection of H_2_O_2_ in situ, chlorophyll and H_2_O_2_ content, qRT-PCR, transcriptome, and proteome analysis.

### 4.2. Determination of Zn Concentration

The shoots and roots of *M. cordata* were collected and washed; in particular, the whole roots were immersed in 25 mmol·L^−1^ EDTA-Na_2_ solution for 10 min and then washed with distilled water again. Before being dried in an air circulation oven at 70 °C, the samples were weighed to obtain the fresh weight and the dry weight of the roots and shoots, respectively. Subsequently, about 0.2 g of the dried samples were then digested following the procedure described by Zhang et al. [20]. An ICP-OES (Optima 8000, PerkinElmer, Waltham, MA, USA) was used to analyze the contents of Zn in *M. cordata.* Zn concentration was calculated on a fresh weight basis (μmol g^−1^FW).

### 4.3. Histochemical Detection of H_2_O_2_

For the histochemical detection of H_2_O_2_ in leaves, the 3,3′-diaminobenzidine (DAB) method was used, following the procedure described by Zhang et al. [71]. The second-youngest leaves were cut and immersed in a 1 mg·mL^−1^ solution of DAB (pH 3.8), vacuum-infiltrated for 10 min, and then incubated at room temperature for 4 h in the dark. Subsequently, the leaves were bleached in boiling ethanol, and images were captured with a Nikon D7100 digital camera.

### 4.4. Determination of Chlorophyll Content

Chlorophyll was extracted from 1.0 g of the fresh leaves (the second-youngest leaves) of *M. cordata* according to the method of Arnon [72]. The chlorophyll content was calculated on a fresh weight (FW) basis (mg g^−1^ FW).

### 4.5. Microscopic Observation of Mesophyll Cells

To detect the cellular characteristics of mesophyll tissue, the second-youngest leaves were cut into small pieces and immersed in FAA fixative solution (Gefan Biotech, Shanghai, China) for more than 24 h. After ethanol dehydration at room temperature, the samples were embedded in paraffin blocks, and 15 μm-thick sections were prepared according to the method of Maniou et al. [73]. The sections were stained following Johansen’s safranin and the fast green protocol [74]. Microscopic images were collected and analyzed. The cytoderm, with lignification, appeared red, and the cellulose cell wall appeared green.

### 4.6. Transcriptome Sequencing

The total RNA was extracted using a FastPure Plant Total RNA Isolation kit (Vazyme, Nanjing, China) according to the method of Zhang et al. [75]. After passing the library inspection, high-throughput sequencing was then performed using a HiSeq 2000 sequencing platform at Genepioneer Biotech (Nanjing, China). The transcriptome sequencing data of the *M. cordata* leaves are shown in Table S1, and 104.02 Gb of clean data were obtained after the raw data were filtered. The sequenced reads were assembled with Trinity software, and 116,944 transcripts and 58,583 unigenes were obtained. With NCBI Blast, the sequences of unigenes were compared with the genome sequence of *M. cordata* (www.ncbi.nlm.nih.gov/nuccore/MVGT01004176, accessed on 15 January 2021) provided by Liu et al. [76], and the annotations of unigenes were obtained using the NCBI (nr), Swiss-prot, GO, COG, KOG, and KEGG databases. The Benjamini–Hochberg correction method was used to adjust the *p*-values (padj) and decrease the number of false positives in the final analysis. Padj < 0.05 and |log2(Fold change)| > 1 were used to determine the significant differential expression between the Zn treatments and the control. When the value of log2(Fold change) was greater than 1, a differentially expressed gene (DEG) was upregulated under the Zn treatment, whereas when the value was less than 1, a DEG was downregulated.

### 4.7. Quantitative Real-Time PCR

Leaf RNA was extracted using a FastPure Plant Total RNA Isolation kit (Vazyme) and reverse transcribed using Hifair^TM^ II 1st Strand cDNA Synthesis for qRT-PCR (Yeasen, Shanghai, China) according to the manufacturer’s instructions. The primers were designed online (https://sg.idtdna.com/PrimerQuest/Home/Index, accessed on 10 July 2021) according to the cds from the *M. cordata* transcriptome (Table S2). The Bio-Rad CFX System and Hifair III One-Step qRT-PCR SYBR Green kit (Yeasen, Shanghai, China) were used for qRT-PCR analysis. The specificity of the amplified PCR products was verified via melting curve analysis, and the reference gene is the Mc18s gene of *M. cordata*.

### 4.8. Proteome Analysis

The leaf proteins of *M. cordata* were extracted using the method of Zhang et al. [55], and the amount of protein was determined using a Bradford Protein Assay kit (Chemstan, Wuhan, China). Approximately 500 μg of proteins were dissolved in a lysis solution with 50 mM Tris-HCl (pH 8.0) with 8 M urea and 1 M dithiothreitol. Subsequently, proteomic analysis was performed using liquid chromatography–tandem mass spectrometry (LC-MS/MS) based on label-free quantification according to the method of Duan et al. [77]. The MS/MS raw data were searched against the *M. cordata* transcriptome database using Proteome Discoverer software (v2.1; Thermo Fisher Scientific, Waltham, MA, USA). Significantly differentially expressed proteins (DEPs) were those with minimum cutoff between the Zn and control treatments of 1.5-fold (up) or 0.67-fold (down) change and significant *t*-test (*p* < 0.05).

### 4.9. Statistical Analysis

The data were analyzed using SPSS 25.0, and the figures were prepared with GraphPad Prism 9. The data are expressed as the mean ± SE (standard error) of three independent replicates; the means denoted by different letters are significantly different (*p* < 0.05, Duncan’s test). The staining experiments were repeated at least five times, with similar results.

## 5. Conclusions

**Figure 10:**
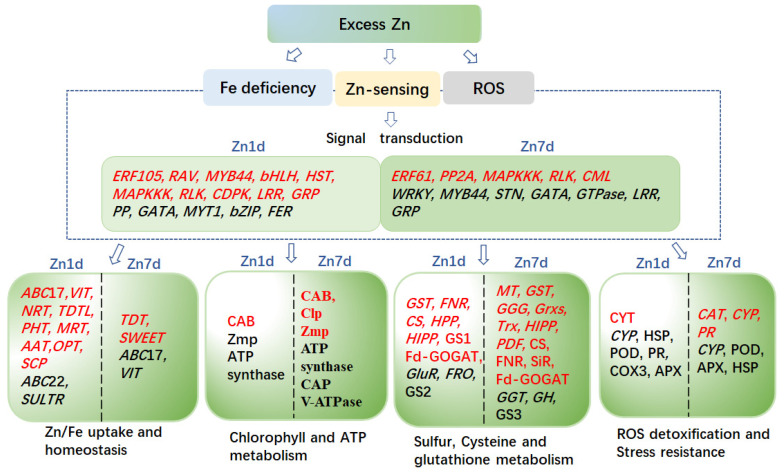
Schematic model of the genes and proteins proposed to mediate Zn tolerance and accumulation mechanisms in the leaves of *Macleaya cordata*. Genes (*Italics*) or proteins in red font were upregulated, whereas those in black font were downregulated by excess Zn. The differentially expressed genes or proteins on the left of the scatter line are identified under the 1-day ZN treatment (Zn 1d), and the right is identified under the 7-day Zn treatment (Zn 7d).

**Figure 11:**
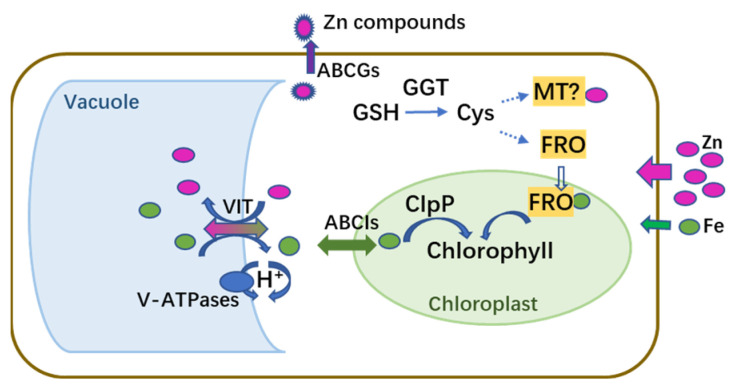
Schematic model of genes and proteins proposed to mediate homeostasis of Zn and Fe in leaf cells of *Macleaya cordata* exposed to excess Zn. Magenta and green circles indicate Zn and Fe, respectively.

Comparative analyses of the transcriptomes and proteomes indicated that *M. cordata* had multiple mechanisms for Zn accumulation and tolerance, as illustrated in the schematic model in Figure 10. It was hypothesized that excess Zn induced ROS production and Fe deficiency, which activated a series of signal molecules in *M. cordata* to cope with those stresses. Fe-deficiency-induced genes, including *VIT*, *ABCIs*, *ABCGs*, and *FRO*, were upregulated in the Zn 1d or 7d treatments, could be responsible for Zn-Fe homeostasis in the cytoplasm and chloroplasts. Fe-deficiency-induced proteins and three V-type ATPases were upregulated in the Zn 7d treatment, which can be responsible for H^+^ homeostasis in the cytoplasm. Moreover, the DEPs of CAB5, ClpP3, and Zmp were upregulated in the Zn 1d treatment and thus could play pivotal roles in chlorophyll synthesis and increase the numbers of mesophyll cells in the leaves of *M. cordata* in the Zn 7d treatment (Figure 11). Therefore, the proteins involved in Zn-Fe homeostasis might be key to Zn tolerance and accumulation in *M. cordata*.

## Data Availability

All of the datasets presented in this study are included in the article and Supplementary Material. The raw datasets generated during the current study are available in ProteomeXchange Consortium (http://proteomecentral.proteomexchange.org, accessed on 21 November 2022) via the iProX partner repository with the project PXD038219.

## Supplementary Materials

The following supporting information can be downloaded at: https://www.mdpi.com/article/10.3390/plants12122275/s1, Table S1: Transcriptome sequencing data of *Macleay cordata* leaves under Zn treatment; Table S2: Primers of some genes for quantitative real-time PCR.
