# Supplementary material for: Response and Tolerance of Macleaya cordata to Excess Zinc Based on Transcriptome and Proteome Patterns
Source: Plants (Basel). 2023 Jun 11;12(12):2275. doi: 10.3390/plants12122275 (PMC10305277; doi:10.3390/plants12122275)
Supplement: Supplementary file 1 [file plants-12-02275-s001.zip › plants-2387681-supplementary.pdf]

Supplementary Materials

Article

Response and tolerance of *Macleaya cordata* to excess zinc based on transcriptome and proteome patterns

Hongxiao Zhang[1]*, Linfeng Hu[2], Xinlong Du[1], Assar Ali Shah[3], Baseer Ahmad[3], Liming Yang[3], Zhiying Mu[4]*

[1] College of Agriculture, Henan University of Science and Technology, Luoyang, China; hxzhang@haust.edu.cn; dxl200004@sina.com
[2] College of Biotechnology, Tianjin University of Science and Technology, Tianjin, China; henryhu391@gmail.com
[3] College of Life Sciences, Nanjing Forestry University, Nanjing, China; assaralishah@yahoo.com; dr.baseerahmadkhan@gmail.com; yangliming@njfu.edu.cn
[4] College of Forestry and Biotechnology, Zhejiang Agriculture and Forestry University, Hangzhou, China; zhiyingmu6@163.com
* Correspondence: hxzhang@haust.edu.cn; zhiyingmu2023@sina.com

Table S1. Transcriptome sequencing data of *Macleay cordata* leaves under Zn treatment.

Samples	Read Number	Base Number	GC Content	%≥Q30
CK-1	24079770	7223931000	43.52	92.78
CK-2	22351713	6705513900	44.08	93.72
CK-3	18952705	5685811500	43.34	95.08
Zn 1d-1	22030032	6609009600	44.36	95.15
Zn 1d-2	23837633	7151289900	44.75	95.20
Zn 1d-3	21502239	6450671700	44.11	94.77
Zn 7d-1	22848084	6854425200	44.31	94.72
Zn 7d-2	21969066	6590719800	44.78	94.89
Zn 7d-3	21566127	6469838100	43.71	94.78

Table S2. Primers of some genes for quantitative real-time PCR in *Macleay cordata* leaves under Zn treatment.

Primer name	Sequence (From 5′ to 3′)
Mc18S-F	CTTCGGGATCGGAGTAATGA
Mc18S-R	GCGGAGTCCTAGAAGCAACA
McABCX1-F	CGGTCGCAGACAACATAAGA
McABCX1-R	AAGGTCAGCAAGGTTGAGTAG
McABCX3-F	AGTGGCAGTGGCAAATCA
McABCX3-R	AGGGAGAGAACATCGAGATCA
McTDT-F	CCTCGTTCTTGGGAGCTTTATC
McTDT-R	TAGAGGCTCCACGCAGAATA
McVIT-F	GGTCTCCACAGCATCTTTGA
McVIT-R	ACTACAAGCTCCGGCTACTA
McMT3c-F	TGTTCTTCTGCTCCAACGTC
McMT3c-R	GTGAGGAAGGAGAGCCAATATG
McWRKY-F	TGGAGAAAGCCCTGAATTGG
McWRKY-R	AAGATGAAGACGGGTTGAGATG
McMYB-F	GGAATCTGACCCACCAACAA
McMYB-R	AACCCAGCAGGAACATTCTC
McERF105-F	TGGACGTCTATTTGGGAGAATG
McERF105-R	GATGACCATCAGCTGGGAATAA
McERF61-F	TTATGATCGTGCGGCGTATAA
McERF61-R	AAGCCTCGAGTTGTCTCTAAAC
